# Self-harming behaviour in liaison psychiatry : Case series and literature review

**DOI:** 10.1192/j.eurpsy.2023.1624

**Published:** 2023-07-19

**Authors:** K. BENALLEL, W. MANSOURI, M. KADIRI

**Affiliations:** psychiatry, Military Hospital Mohammed V - Rabat, Rabat, Morocco

## Abstract

**Introduction:**

Self-harm (SH) is common, in particular among young people. It can be seen in a wide range of psychiatric disorders, ranging from anti-social personality disorders to schizophrenia and mood disorders. In the extreme, self-harm can be functionally life-threatening. Such is the case of phlebotomy, emasculations and self-amputation. The severity of certain damage and the urgency of an initial somatic treatment contribute to make self-harm one of the most frequent reasons for intervention in liaison psychiatry.

**Objectives:**

Through our case series and a literature review, we tried to describe the socio-demographic and psychopathological characteristics of the self-harmers and to identify the specificities of their management in liaison psychiatry.

**Methods:**

It is a descriptive cross-sectional study, in the psychiatric department of a general hospital in Rabat, concerning patients evaluated for SH with or without other psychiatric manifestations. The data collected are analysed using the statistical software ‘JAMOVI’. Patients seen in psychiatric consultations, in medical-surgical emergencies or in liaison psychiatry for SH were included. Patients already hospitalized in psychiatry were excluded.

**Results:**

35 patients were recruted of who 65.7% were male. 68.6% were single. 51.4% had a low socio-economic level and 42.9% had an average level. 48.6% had a psychiatric history of which 22.9% had attempted suicide. Abuse was present in 34.3%, family separation in 22.9%, death of a parent in 20% while no patient reported sexual abuse. The most common method used was a razor blade in 57.1% of cases. The most mutilated site was the forearms in 65.7%, following a frustration in 60% and a conflict situation in 25.7%. 48.6% were hospitalised (34.5% in psychiatry, 5.6% in intensive care and 5.6% in otorhinolaryngology).

**Conclusions:**

Self-harm is a frequent pathological behaviour whose incidence is increasing. Understanding the psychological and biological basis of self-harm will help to improve the management of these patients and prevent the recurrence of this dangerous behavior and its complication by suicide.

**Disclosure of Interest:**

None Declared

